# Supplementary material for: Differential Gene Expression Associated with Soybean Oil Level in the Diet of Pigs
Source: Animals (Basel). 2022 Jun 25;12(13):1632. doi: 10.3390/ani12131632 (PMC9265114; doi:10.3390/ani12131632)
Supplement: Supplementary file 1 [file animals-12-01632-s001.zip › animals-1734763-supplementary/figures S1-16.pdf]

(A)

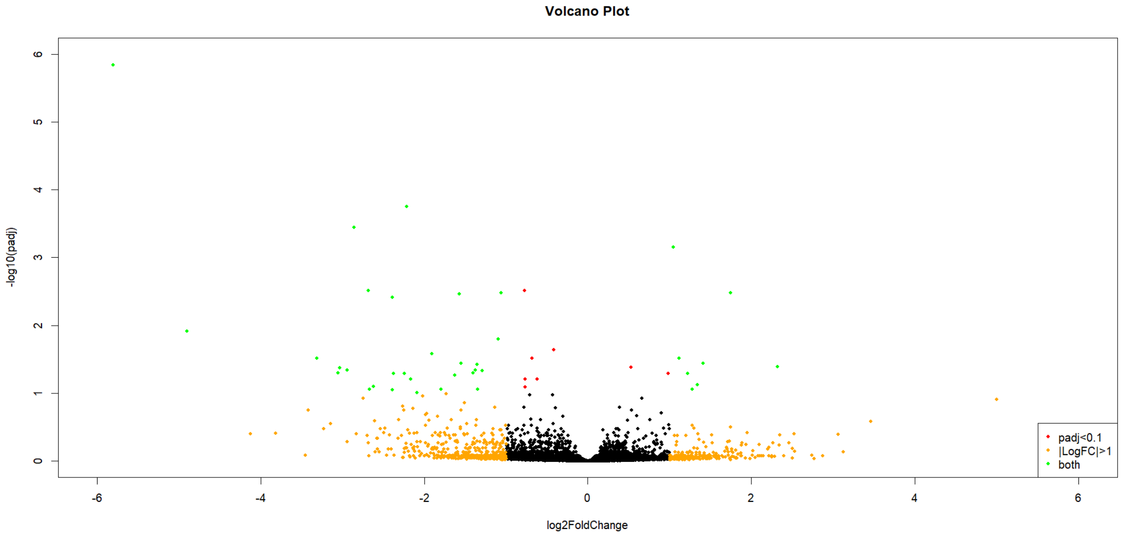
Volcano Plot
-log10(padj)
0
1
2
3
4
5
6
-6
-4
-2
0
2
4
6
log2FoldChange
padj<0.1
|LogFC|>1
both

(B)

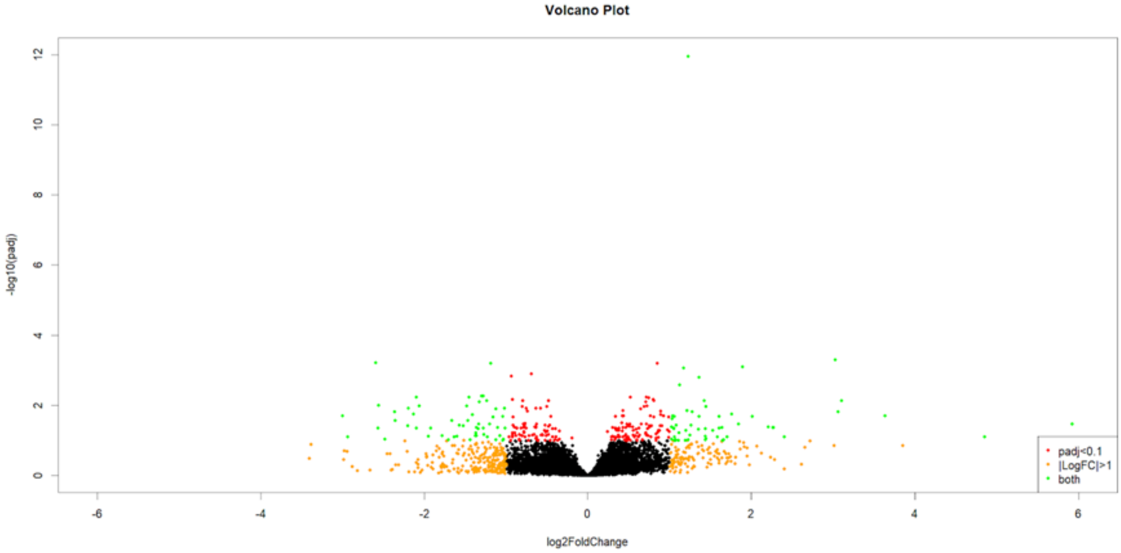
Volcano Plot
-log10(padj)
0
2
4
6
8
10
12
-6
-4
-2
0
2
4
6
log2FoldChange
padj<0.1
|LogFC|>1
both

(C)

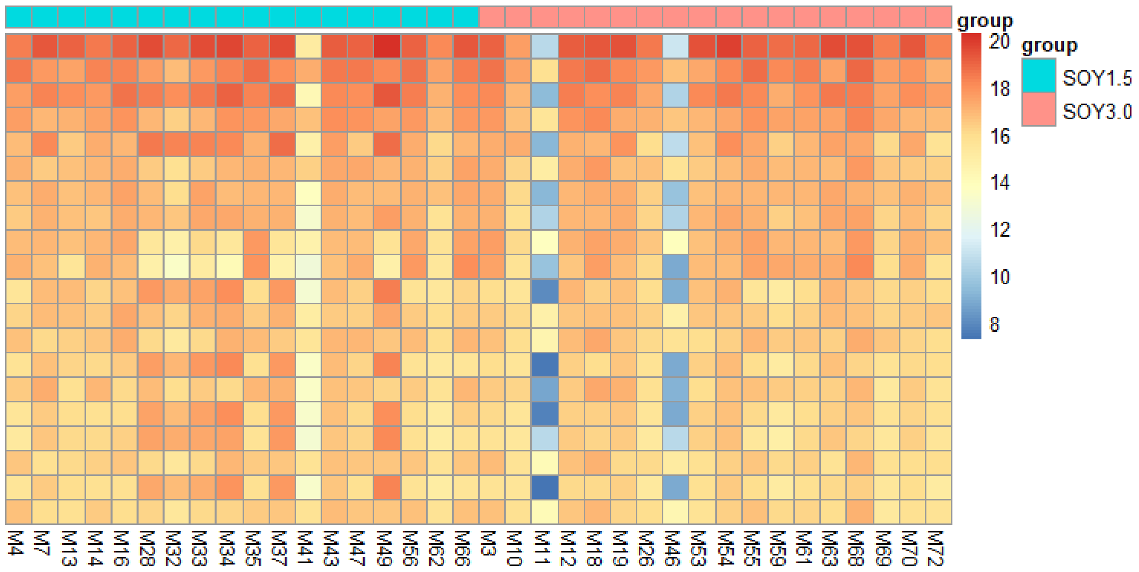


(D)

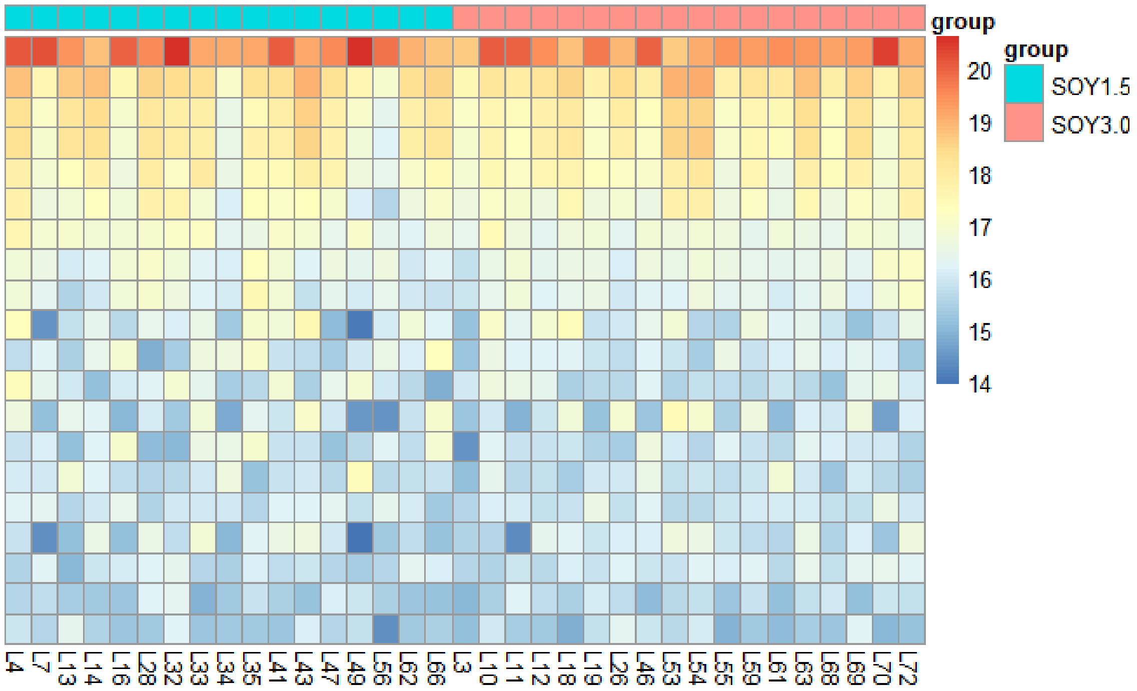


Figure S1. Volcano plot of log2 fold change (x-axis) versus -log10FDR-corrected p-value in RNA-Seq data from (A) skeletal muscle and (B) liver tissue and Heatmap of the count matrix using transformed data for skeletal muscle (C) and liver (D) of pigs fed with two different level of soybean oil in the diet (1.5 % and 3.0 % of soybean oil).

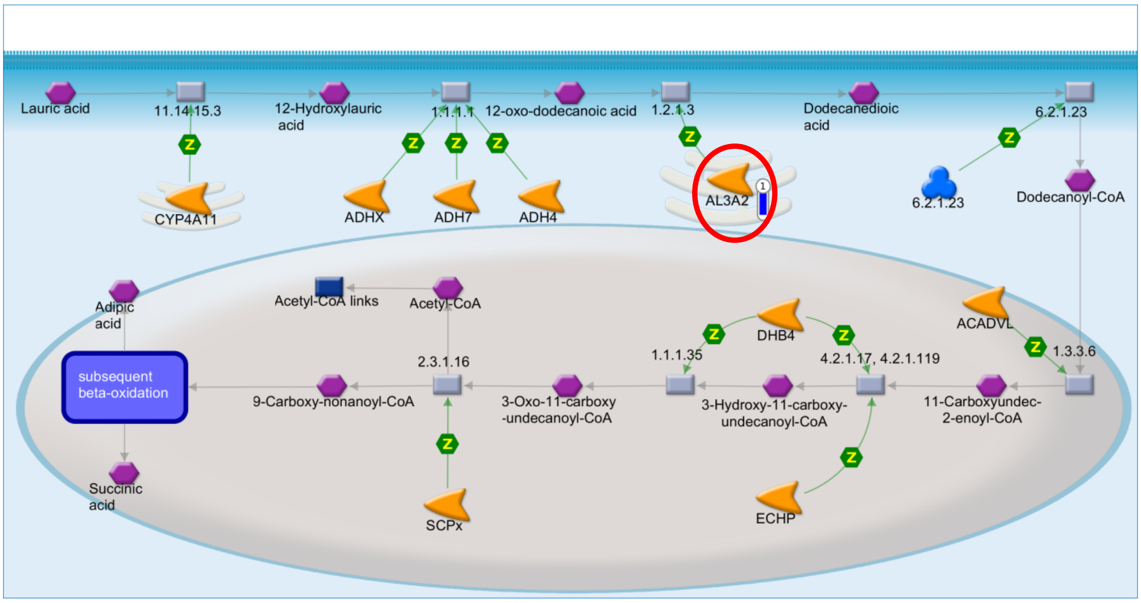


Figure S2: Fatty Acid Omega Oxidation pathway map created by using MetaCore software (*p*-value <0.10) and the list of differentially expressed genes (FDR 10%) in the skeletal muscle of fed with two different levels of soybean oil in the diet (1.5 % and 3.0 % of soybean oil). The blue thermometer indicates that the DEG is down-regulated (log2 fold change -0.77) in the diet with 1.5 % of soybean oil (SOY1.5). Green arrows indicate positive interaction and gray arrows indicates unspecified interaction. The DEG is highlighted with a red circle. For a detailed definition, see https://portal.genego.com/legends/MetaCoreQuickReferenceGuide.pdf.

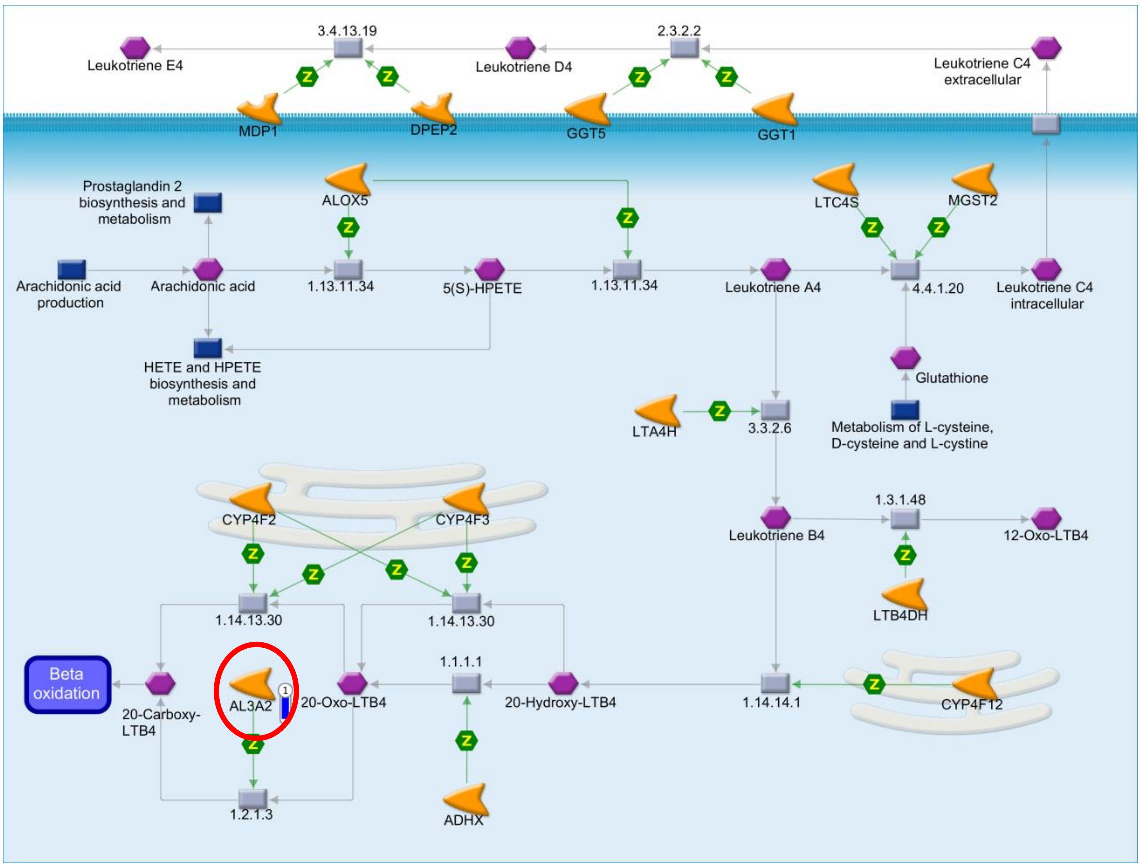


Figure S3. Leukotriene 4 biosynthesis and metabolism pathway map created by using MetaCore software ($p$-value < 0,1) and the list of differentially expressed genes (FDR 10%) in the skeletal muscle of pigs fed with two different levels of soybean oil in the diet (1.5 % and 3.0 % of soybean oil). The blue thermometer indicates that the DEG is down-regulated (log2foldchange -0.77) in the diet with 1.5 % of soybean oil (SOY1.5). Green arrows indicate positive interaction and gray arrows indicates unspecified interaction. The DEG is highlighted with a red circle. For a detailed definition, see https://portal.genego.com/legends/MetaCoreQuickReferenceGuide.pdf.

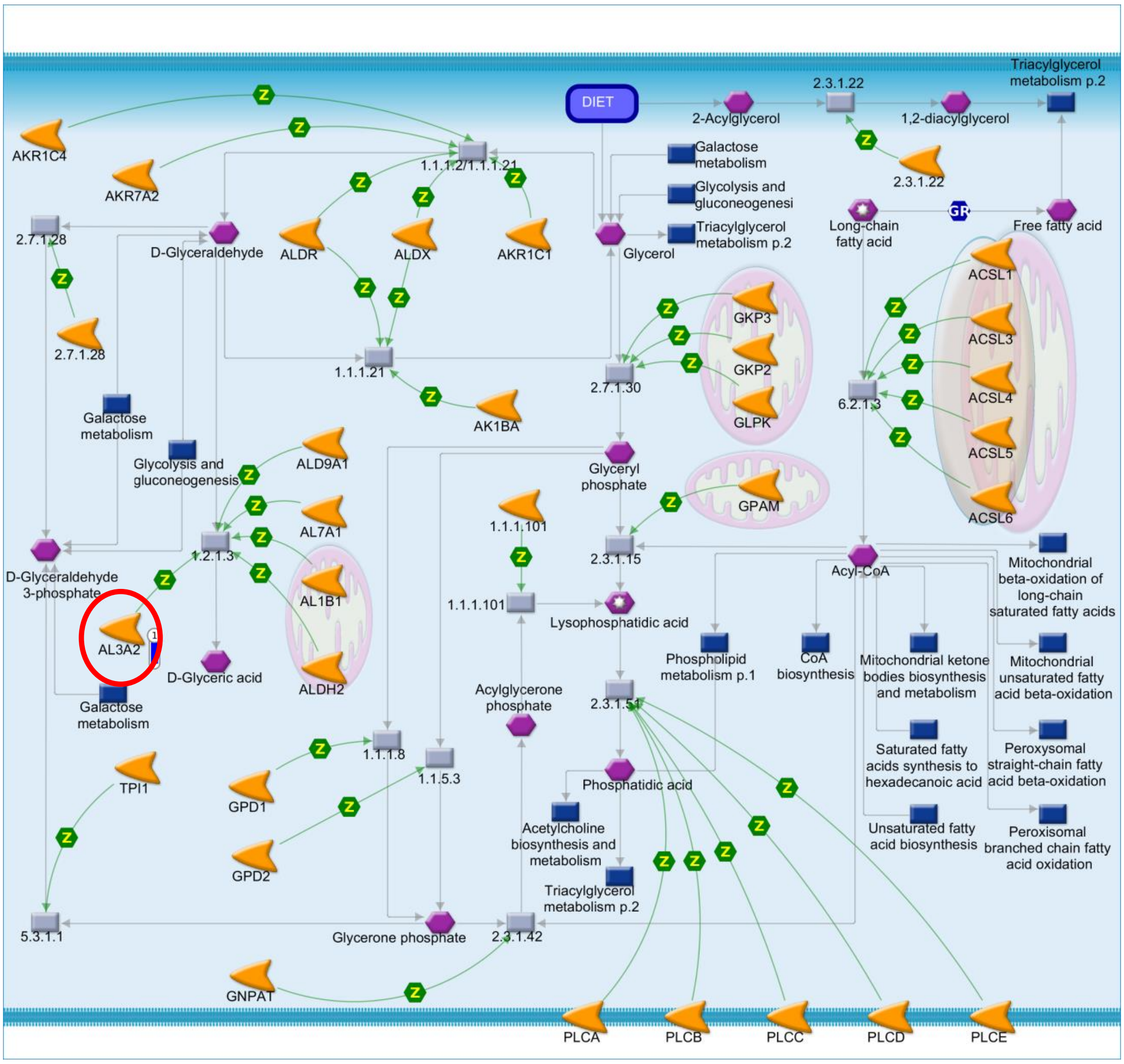


Figure S4. Triacylglycerol metabolism p.1 pathway map created by using MetaCore software ( *p*-value < 0,1) and the list of differentially expressed genes (FDR 10%) in the skeletal muscle of pigs fed with two different levels of soybean oil in the diet (1.5 % and 3.0 % of soybean oil). The blue thermometer indicates that the DEG is down-regulated (log2foldchange -0.77) in the diet with 1.5 % of soybean oil (SOY1.5). Green arrows indicate positive interaction and gray arrows indicates unspecified interaction. The DEG is highlighted with a red circle. For a detailed definition, see https://portal.genego.com/legends/MetaCoreQuickReferenceGuide.pdf.

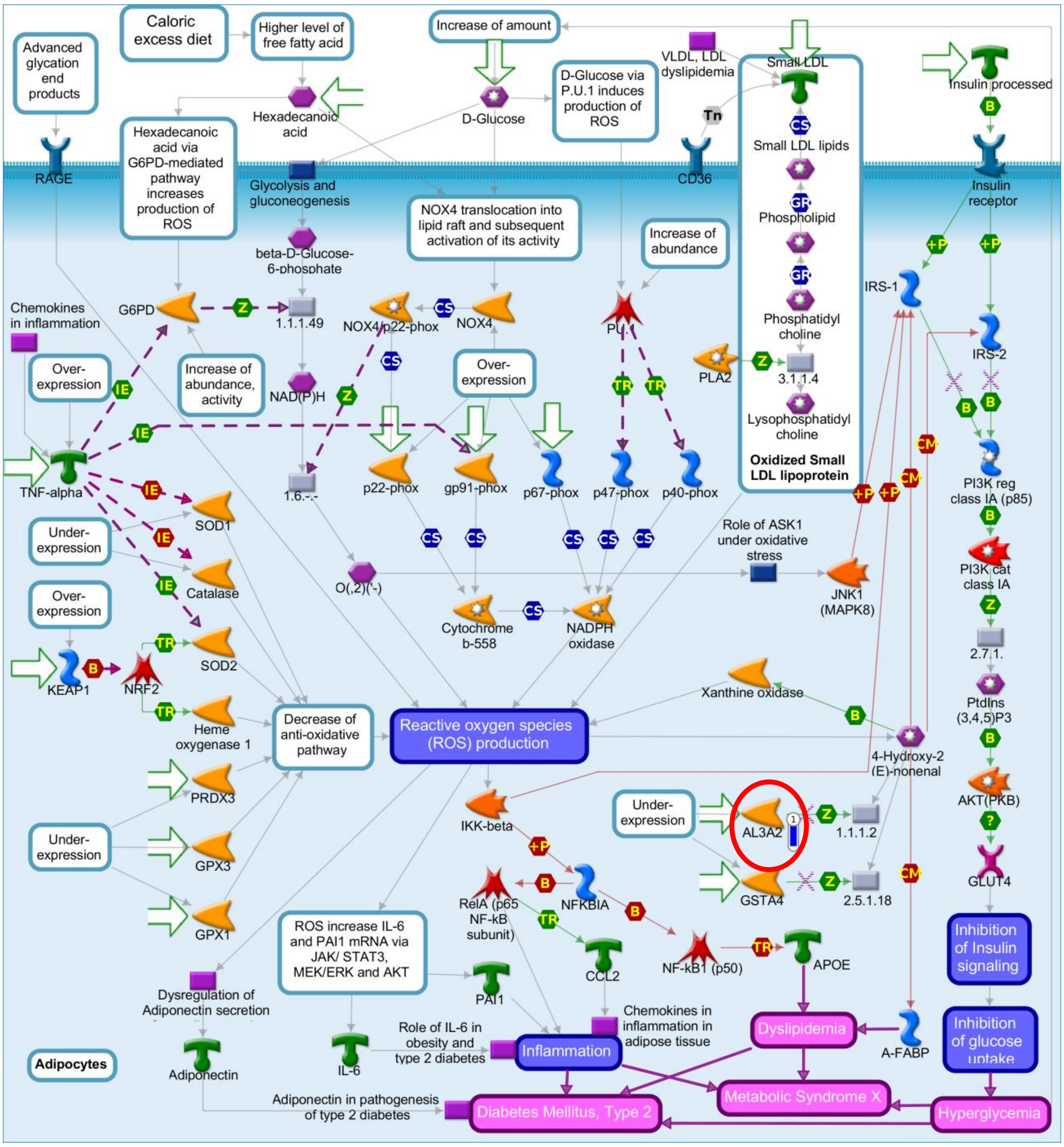


Figure S5. Oxidative stress in adipocyte dysfunction in type 2 diabetes and metabolic syndrome X pathway map created by using MetaCore software ($p$-value <0.1) and the list of differentially expressed genes (FDR 10%) in the skeletal muscle of pigs fed with two different levels of soybean oil in the diet (1.5 % and 3.0 % of soybean oil). The blue thermometer indicates that the DEG is down-regulated (log2 fold change -0.77) in the diet with 1.5 % of soybean oil (SOY1.5). A green arrow indicates positive interactions; a red arrow indicates negative interaction and gray arrows indicates unspecified interaction. The DEG is highlighted with a red circle. For a detailed definition, see https://portal.genego.com/legends/MetaCoreQuickReferenceGuide.pdf.

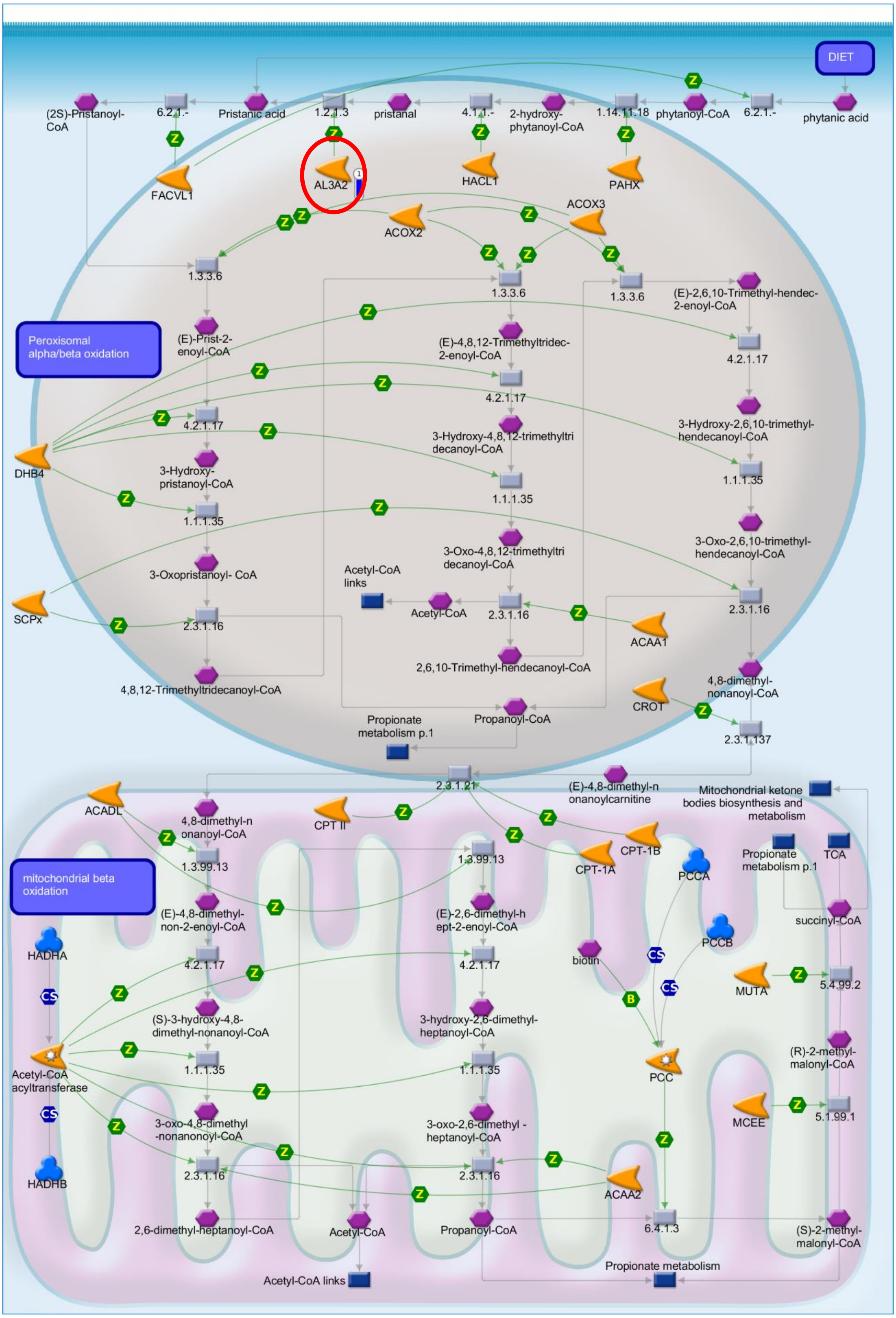


Figure S6. Peroxisomal branched chain fatty acid oxidation pathway map created by using MetaCore software (*p*-value <0.1) and the list of differentially expressed genes (FDR 10%) in the skeletal muscle of pigs fed with two different levels of soybean oil in the diet (1.5 % and 3.0 % of soybean oil). The blue thermometer indicates that the DEG is down-regulated (log2 fold change

-0.77) in the diet with 1.5 % of soybean oil (SOY1.5). A green arrow indicates positive interaction and gray arrows indicates unspecified interaction. The DEG is highlighted with a red circle. For a detailed definition, see https://portal.genego.com/legends/MetaCoreQuickReferenceGuide.pdf.

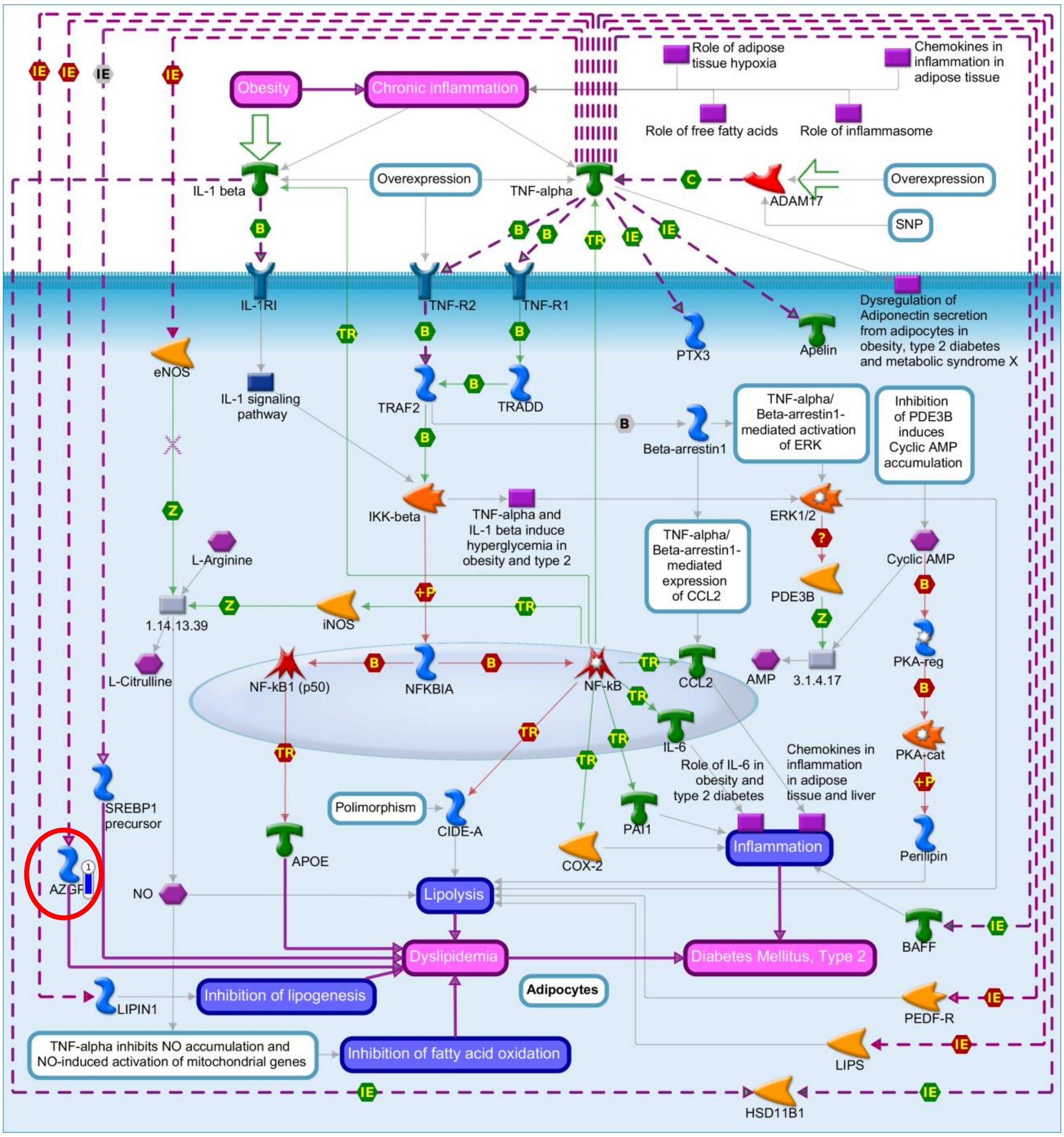


Figure S7. *TNF-alpha, IL-1 beta* induces dyslipidemia and inflammation in obesity and type 2 diabetes in adipocytes pathway map created by using MetaCore software ($p$-value <0.10) from the list of differentially expressed genes (FDR 10%) in the skeletal muscle of pigs fed with two different levels of soybean oil in the diet (1.5 % and 3.0 % of soybean oil). The blue thermometer indicates that the DEG is down-regulated (log2 fold change -2.67) in the diet with 1.5 % of soybean oil (SOY1.5). Purple lines indicates enhances in diseases and purple dotted line emerges in diseases. Green arrows indicate positive interactions; red arrows indicate negative interaction and gray arrows indicates unspecified interaction. The DEG is highlighted with a

red circle. For a detailed definition, see https://portal.genego.com/legends/MetaCoreQuickReferenceGuide.pdf.

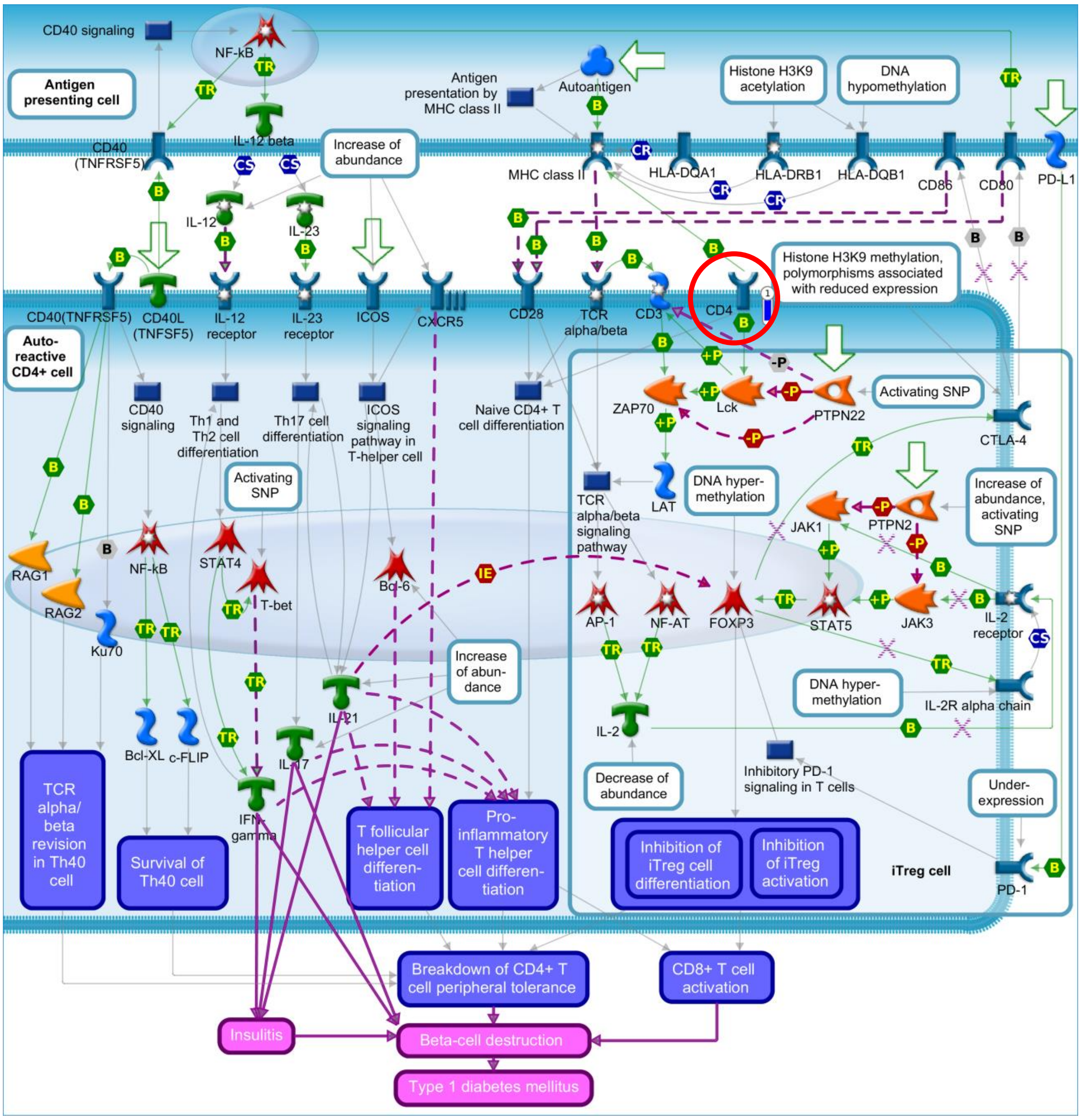


Figure S8. Breakdown of CD4+ T cell peripheral tolerance in type 1 diabetes mellitus pathway map created by MetaCore software (*p*-value <0.10) from the list of differentially expressed genes (FDR 10%) in the skeletal muscle of pigs fed with two different levels of soybean oil in the diet (1.5 % and 3.0 % of soybean oil). The blue thermometer indicates that the DEG is down-regulated (log2 fold change -1.57) in the diet with 1.5 % of soybean oil (SOY1.5). Purple lines indicates enhances in diseases and purple dotted line emerge in diseases. A green arrow indicates positive interactions; red arrows indicate negative interaction and gray arrows indicates unspecified interaction. The DEG is highlighted with a red circle. For a detailed definition, see https://portal.genego.com/legends/MetaCoreQuickReferenceGuide.pdf.

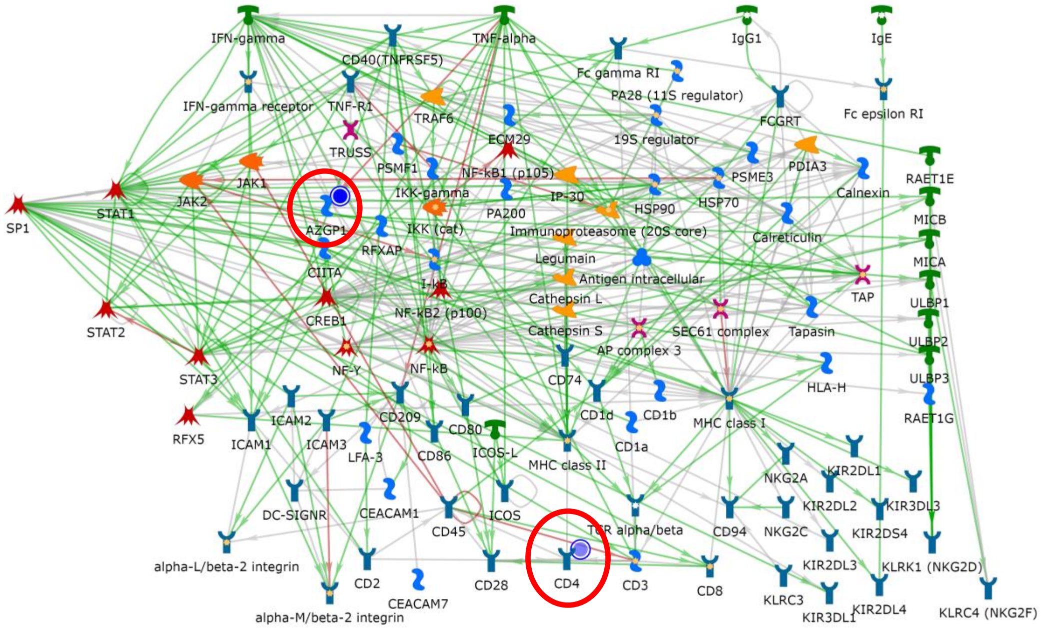


Figure S9. Process network Immune response antigen presentation identified by MetaCore software using the list of differentially expressed genes (FDR 10%) in the skeletal muscle of pigs fed with two different levels of soybean oil in the diet (1.5 % and 3.0 % of soybean oil). Green arrows indicate positive interactions, a red arrow indicates negative interactions, and a gray arrow indicates unspecified interactions. The DEG is highlighted with a red circle. For a detailed definition, see https://portal.genego.com/legends/MetaCoreQuickReferenceGuide.pdf.

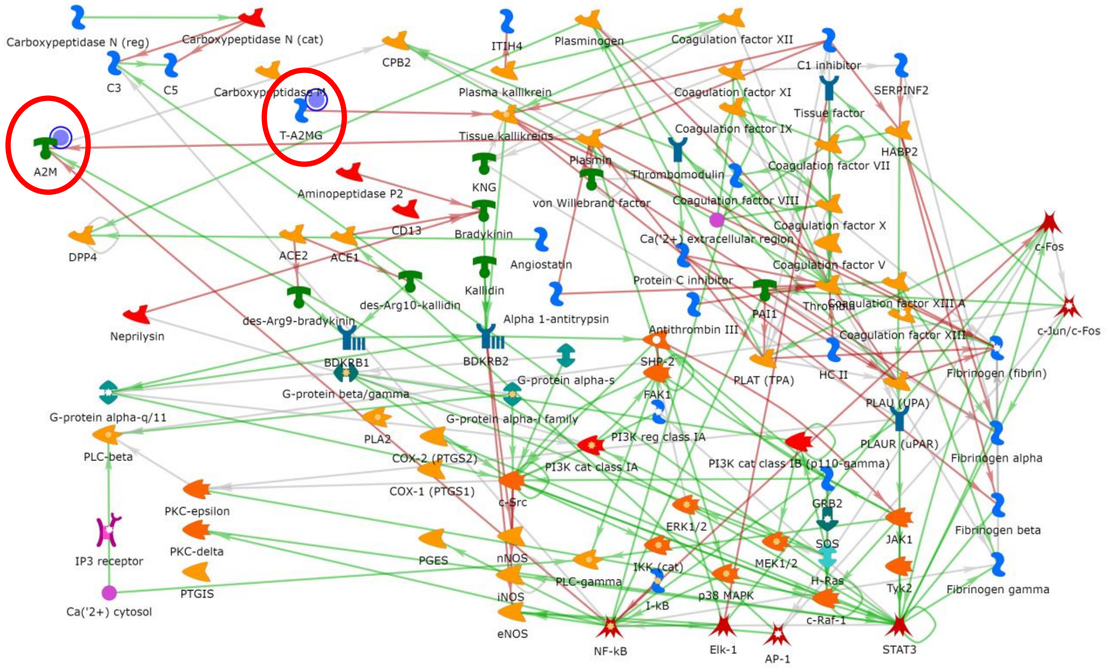


Figure S10. Process network Inflammation_Kallikrein-kinin system identified by MetaCore software using the list of differentially expressed genes (FDR 10%) in the skeletal muscle of pigs

fed with two different levels of soybean oil in the diet (1.5 % and 3.0 % of soybean oil). Green arrows indicates positive interactions, red arrows indicates negative interactions and gray arrows indicates unspecified interactions. The DEG is highlighted with a red circle. For a detailed definition, see https://portal.genego.com/legends/MetaCoreQuickReferenceGuide.pdf

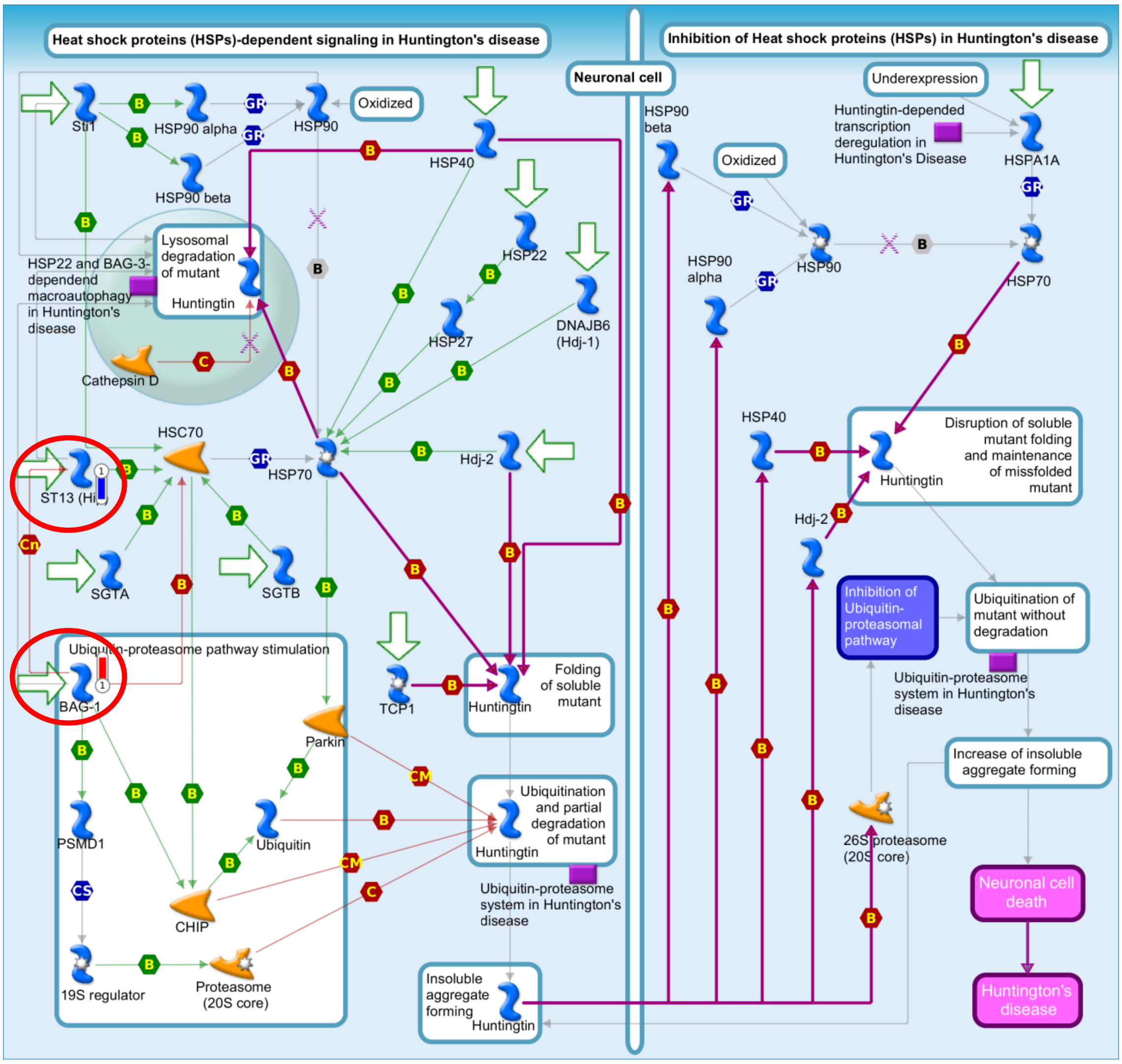


Figure S11. HSP70 and HSP40-dependent folding in Huntington's disease pathway map created by MetaCore software (*p*-value <0.10) and the list of differentially expressed genes (FDR 10%) in the liver of male pigs fed with two different levels of soybean oil in the diet (1.5 % and 3.0 % of soybean oil). The blue thermometer indicates that the DEG is down-regulated (log2 fold change -0.35) and the red thermometer indicates that the DEG is up-regulated (log2 fold change +0.35) in the diet with 1.5 % of soybean oil (SOY1.5). Purple lines indicates enhances in diseases and purple dotted line emerges in diseases. Green arrows indicate positive interactions, red arrows indicate negative interactions, and a grey arrow indicates unspecified interactions. The DEG is highlighted with a red circle. For a detailed definition, see https://portal.genego.com/legends/MetaCoreQuickReferenceGuide.pdf.

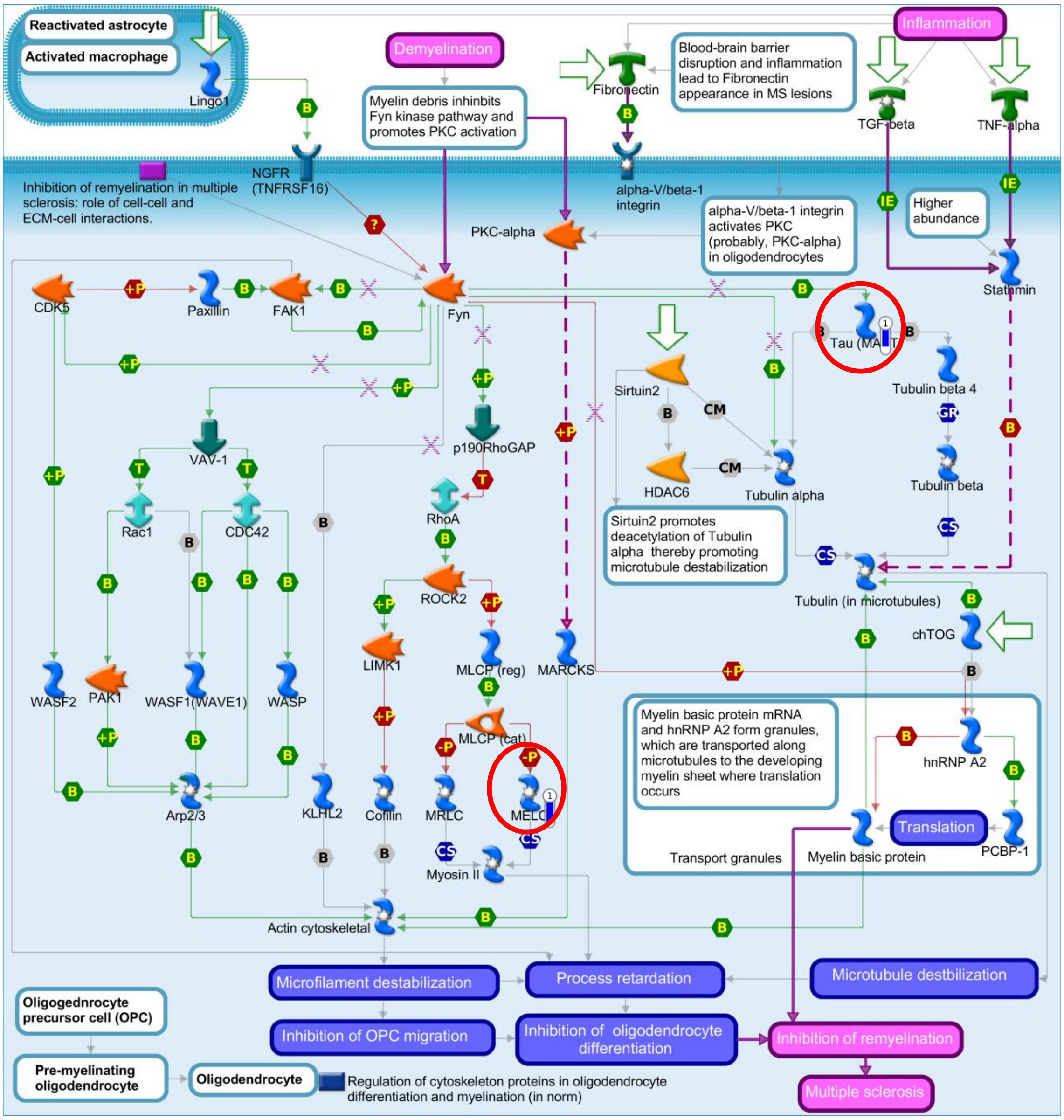


Figure S12. Inhibition of remyelination in multiple sclerosis: regulation of cytoskeleton proteins pathway map created by using MetaCore software ($p$-value <0.10) and the list of differentially expressed genes (FDR 10%) in the liver of pigs fed with two different levels of soybean oil in the diet (1.5 % and 3.0 % of soybean oil). The blue thermometer indicates that the DEG is down-regulated (log2 fold change -1.18 and -1.37) in the diet with 1.5 % of soybean oil (SOY1.5). Purple lines indicates enhances in diseases and purple dotted line emerges in diseases. A green arrow indicates positive interactions, a red arrow indicates negative interactions and grey arrows indicate unspecified interactions. The DEG is highlighted with a red circle. For a detailed definition, see https://portal.genego.com/legends/MetaCoreQuickReferenceGuide.pdf.

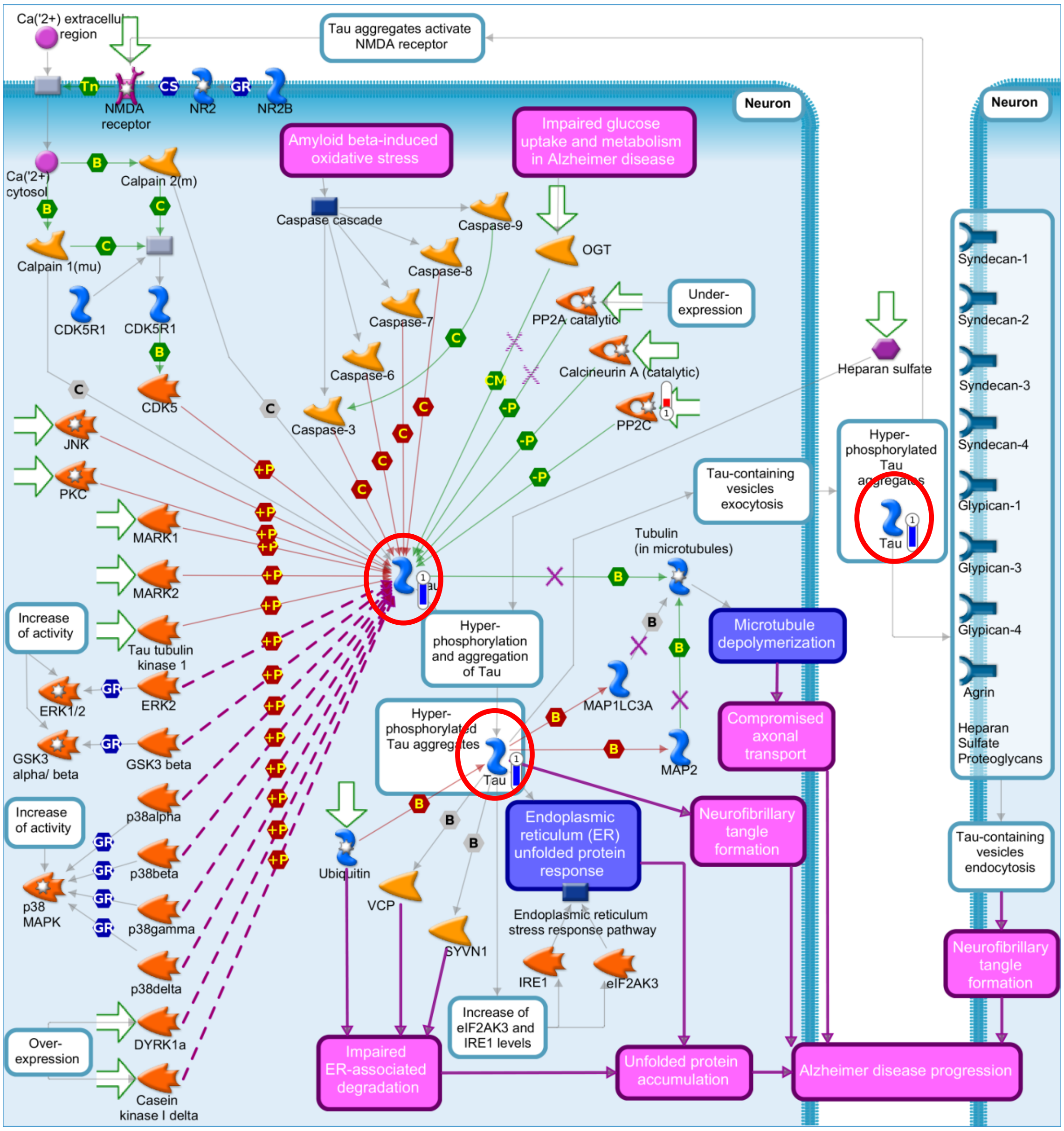


Figure S13. Tau pathology in Alzheimer disease pathway map created by using MetaCore software (*p*-value <0.10) and the list of differentially expressed genes (FDR 10%) in the liver of pigs fed with two different levels of soybean oil in the diet (1.5 % and 3.0 % of soybean oil). The blue thermometer indicates that the DEG is down-regulated (log2 fold change -1.18) and red thermometer indicates that the DEG is up-regulated (log2 fold change +0.42) in the diet with 1.5 % of soybean oil (SOY1.5). Purple lines indicates enhances in diseases and purple dotted line emerges in diseases. Green arrows indicate positive interactions, a red arrow indicates negative interactions and grey arrows indicate unspecified interactions. The DEG is highlighted with a red circle. For a detailed definition, see https://portal.genego.com/legends/MetaCoreQuickReferenceGuide.pdf.

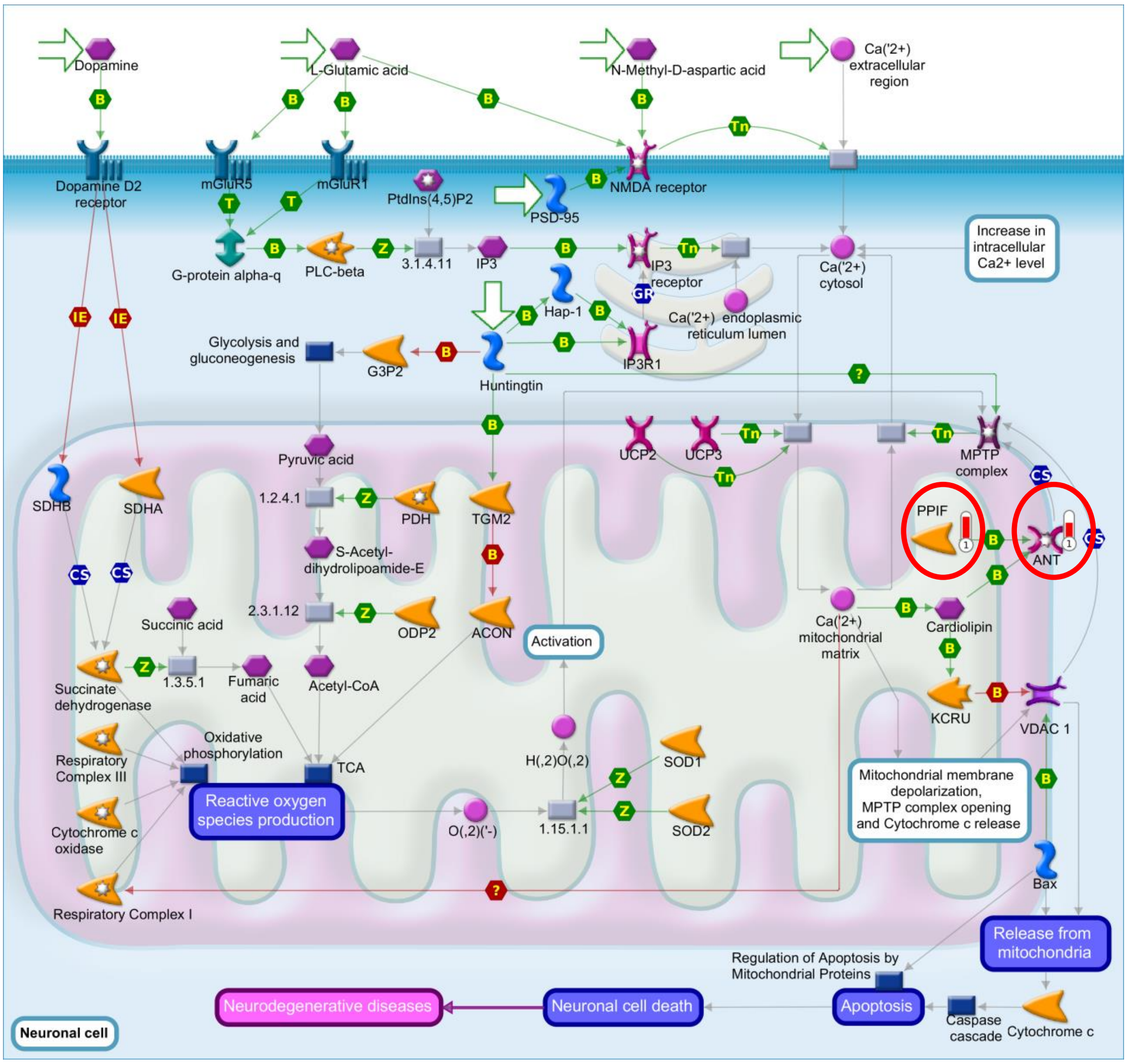


Figure S14. Mitochondrial dysfunction in neurodegenerative diseases pathway map created by using MetaCore software (*p*-value <0.1) and the list of differentially expressed genes (FDR 10%) in the liver of fed with two different levels of soybean oil in the diet (1.5 % and 3.0 % of soybean oil). The red thermometer indicates that the DEG is up-regulated (log2 fold change +0.81 and +0.57) in the diet with 1.5 % of soybean oil (SOY1.5). Purple lines indicates enhances in diseases and purple dotted line emerges in diseases. A green arrow indicates positive interactions, red arrows indicate negative interactions and grey arrows indicate unspecified interactions. The DEG is highlighted with a red circle. For a detailed definition, see https://portal.genego.com/legends/MetaCoreQuickReferenceGuide.pdf.

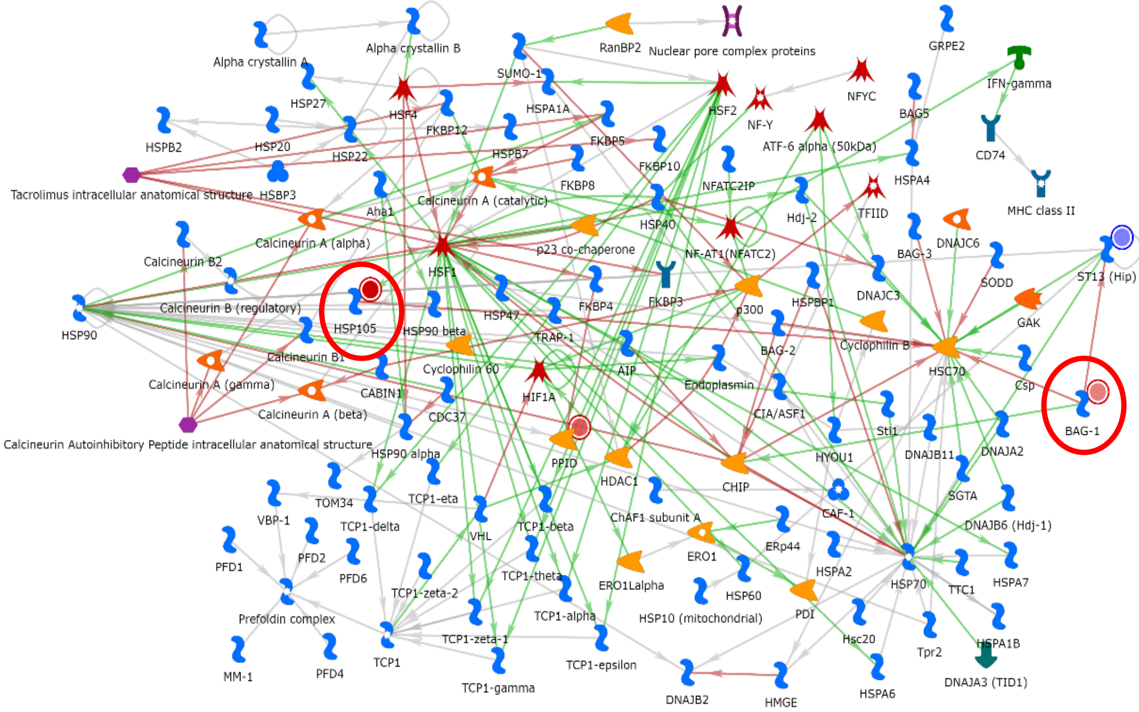


Figure S15. Process network Protein folding in normal condition identified by MetaCore software from the list of differentially expressed genes (FDR 10%) in the liver of pigs fed with two different levels of soybean oil in the diet (1.5 % and 3.0 % of soybean oil). A green arrow indicates positive interactions; red arrows indicate negative interaction and gray arrows indicates unspecified interaction. The DEG is highlighted with a red circle. For a detailed definition, see https://portal.genego.com/legends/MetaCoreQuickReferenceGuide.pdf.

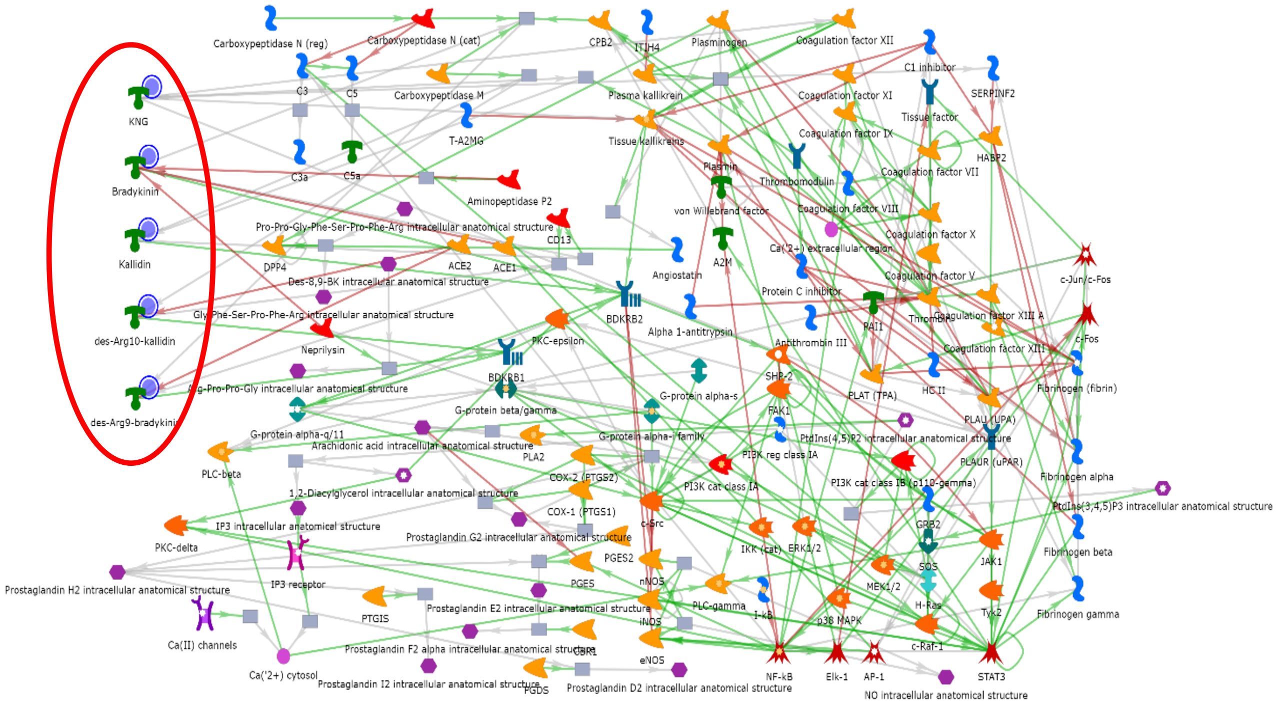

Figure S16. Process network Inflammation_Kallikrein-kinin system identified by MetaCore software from the list of differentially expressed genes (FDR 10%) in the liver of pigs fed with two different levels of soybean oil in the diet (1.5 % and 3.0 % of soybean oil). Green arrows indicate positive interactions; red arrows indicate negative interaction and gray arrows indicates unspecified interaction. The DEG is highlighted with a red circle. For a detailed definition, see https://portal.genego.com/legends/MetaCoreQuickReferenceGuide.pdf.
