# Supplementary material for: Differential Gene Expression Associated with Soybean Oil Level in the Diet of Pigs
Source: Animals (Basel). 2022 Jun 25;12(13):1632. doi: 10.3390/ani12131632 (PMC9265114; doi:10.3390/ani12131632)
Supplement: Supplementary file 1 [file animals-12-01632-s001.zip › animals-1734763-supplementary/Tables_S1-S4.pdf]

Table S1: Composition of the experimental diets (as-fed basis).

Item	Grower I (day 0 to 21)		Grower II (day 21 to 42)		Finisher I (day 42 to 56)		Finisher II (day 56 to 63)		Finisher III (day 63 to 70)		Finisher IV (day 70 to 98)	
	SOY1.5	SOY3.0	SOY1.5	SOY3.0	SOY1.5	SOY3.0	SOY1.5	SOY3.0	SOY1.5	SOY3.0	SOY1.5	SOY3.0
Ingredient, %												
Corn, 7.5% CP[1]	63.47	61.88	66.40	64.71	69.13	67.54	69.63	68.04	69.59	68.00	70.09	68.50
Soybean meal, 46% CP	28.33	28.42	26.10	26.29	23.37	23.46	23.37	23.46	22.93	23.02	22.93	23.02
Meat and bone meal, 44% CP	3.00	3.00	3.00	3.00	3.00	3.00	3.00	3.00	3.00	3.00	3.00	3.00
Fat source	1.50	3.00	1.50	3.00	1.50	3.00	1.50	3.00	1.50	3.00	1.50	3.00
Dicalcium phosphate	0.55	0.56	0.56	0.57	0.26	0.27	0.26	0.27	0.27	0.27	0.26	0.27
Limestone	0.43	0.42	0.38	0.38	0.84	0.84	0.76	0.75	0.84	0.84	0.69	0.69
Salt	0.50	0.50	0.50	0.50	0.50	0.50	0.50	0.50	0.50	0.50	0.50	0.50
Vitamin-mineral premix[2]	1.61	1.61	1.08	1.08	1.01	1.01	0.60	0.60	1.02	1.01	0.66	0.65
L-Lysine.HCl	0.35	0.35	0.29	0.29	0.25	0.25	0.25	0.25	0.20	0.20	0.20	0.20
DL-Methionine	0.11	0.11	0.07	0.08	0.04	0.04	0.04	0.04	0.02	0.02	0.03	0.03
L-Threonine	0.14	0.15	0.11	0.11	0.09	0.09	0.09	0.09	0.06	0.06	0.06	0.07
L-Tryptophan	0.01	0.01	-	-	-	-	-	-	-	-	-	-
Ractopamine.HCl, 2%	-	-	-	-	-	-	-	-	0.08	0.08	0.08	0.08
Calculated composition[3]												
Metabolizable energy, Mcal/kg	3.28	3.36	3.29	3.36	3.28	3.36	3.29	3.36	3.28	3.35	3.29	3.36
SID[4] Lysine, %	1.15	1.15	1.05	1.05	0.95	0.95	0.95	0.95	0.90	0.90	0.90	0.90
SID Methionine + Cysteine, %	0.62	0.62	0.57	0.57	0.51	0.51	0.51	0.51	0.49	0.49	0.49	0.49
SID Threonine, %	0.75	0.75	0.68	0.68	0.63	0.63	0.63	0.63	0.58	0.59	0.59	0.59
SID Tryptophan, %	0.22	0.22	0.20	0.20	0.18	0.18	0.18	0.18	0.18	0.18	0.18	0.18
Calcium, %	0.84	0.84	0.81	0.81	0.80	0.80	0.77	0.77	0.80	0.80	0.75	0.75
Available Phosphorous, %	0.42	0.42	0.42	0.42	0.34	0.34	0.34	0.34	0.34	0.34	0.34	0.34
Analyzed composition, %												
CP	19.71	19.64	18.75	18.71	17.66	17.58	17.66	17.59	17.41	17.33	17.42	17.35
Ether extract	4.49	5.49	4.03	5.53	3.63	6.57	3.63	5.59	3.16	5.61	3.16	5.61

[1]CP = crude protein.
[2]Provided per kilogram of diet: 6.500 UI vitamin A; 1.800 UI vitamin D_3; 30 UI vitamin E; 2 mg vitamin K_3; 1.2 mg vitamin B_1; 3.4 mg vitamin B_2; 2.0 mg vitamin B_6; 125 mg Cu; 80 mg Fe; 40 mg Mn; 0.35 mg Se; 1.25 mg Zn.
[3]Calculated according to Rostagno et al. (2011).
[4]SID = standardized ileal digestible.
Adapted from Almeida et al., 2021.

Table S2: Analyzed fatty acid profile of grower diets (as-fed basis).

	Grower I (day 0 to 21)		Grower II (day 21 to 42)	
Fatty acid, %	SOY1.5	SOY3.0	SOY1.5	SOY3.0
Saturated fatty acid (SFA)				
Myristic acid (C14:0)	ND[1]	1.85	0.24	0.31
Palmitic acid (C16:0)	13.97	12.76	13.49	14.37
Margaric acid (C17:0)	ND	ND	0.15	ND
Stearic acid (C18:0)	4.21	2.44	4.01	4.50
Arachidic acid (C20:0)	0.46	ND	0.48	0.45
Behenic acid (C22:0)	0.33	ND	0.22	0.24
Monounsaturated fatty acid (MUFA)				
Palmitoleic acid (C16:1)	0.30	ND	0.19	0.26
Oleic acid (C18:1 n-9)	28.97	23.27	33.21	31.92
Eicosenoic acid (C20:1 n-9)	0.27	ND	0.28	0.21
Polyunsaturated fatty acid (PUFA)				
Linoleic acid (C18:2 n-6)	47.62	55.65	45.33	44.80
Alpha-linolenic acid (C18:3 n-3)	3.57	5.85	2.41	2.95
Eicosapentaenoic acid (C20:5 n-3)	ND	ND	ND	ND
Docosahexaenoic acid (C22:6 n-3)	ND	ND	ND	ND
Total SFA	18.97	17.05	18.59	19.87
Total MUFA	29.54	23.27	33.68	32.39
Total PUFA	51.19	61.5	47.74	47.75
PUFA:SFA ratio[2]	2.70	3.61	2.57	2.40

[1]ND = not detectable.

[2]PUFA:SFA ratio = total PUFA/total SFA.

Adapted from Almeida et al., 2021.

Table S3: Analyzed fatty acid profile of finisher diets (as-fed basis).

	Finisher I (day 42 to 56)		Finisher II (day 56 to 63)	
Fatty acid, %	SOY1.5	SOY3.0	SOY1.5	SOY3.0
Saturated fatty acid (SFA)				
Myristic acid (C14:0)	0.29	1.87	ND	ND
Palmitic acid (C16:0)	13.62	19.75	11.88	12.59
Margaric acid (C17:0)	ND[1]	0.34	ND	ND
Stearic acid (C18:0)	4.29	4.96	3.13	2.83
Arachidic acid (C20:0)	0.43	0.43	0.42	0.41
Behenic acid (C22:0)	ND	ND	0.32	0.21
Monounsaturated fatty acid (MUFA)				
Palmitoleic acid (C16:1)	0.21	3.49	0.11	0.11
Oleic acid (C18:1 n-9)	35.64	36.44	30.79	34.84
Eicosenoic acid (C20:1 n-9)	ND	ND	0.20	0.23
Polyunsaturated fatty acid (PUFA)				
Linoleic acid (C18:2 n-6)	42.82	27.52	48.90	46.03
Alpha-linolenic acid (C18:3 n-3)	2.70	1.65	4.26	2.76
Eicosapentaenoic acid (C20:5 n-3)	ND	ND	ND	ND
Docosahexaenoic acid (C22:6 n-3)	ND	ND	ND	ND
Total SFA	18.63	27.35	15.75	16.04
Total MUFA	35.85	39.93	31.1	35.18
Total PUFA	45.52	29.17	53.16	48.79
PUFA:SFA ratio[2]	2.44	1.07	3.38	3.04

[1]ND = not detectable.

[2]PUFA:SFA ratio = total PUFA/total SFA.

Adapted from Almeida et al., 2021.

Table S4: Analyzed fatty acid profile of finisher diets (as-fed basis).

	Finisher III (day 63 to 70)		Finisher IV (day 70 to 98)	
Fatty acid, %	SOY1.5	SOY3.0	SOY1.5	SOY3.0
Saturated fatty acid (SFA)				
Myristic acid (C14:0)	0.43	0.28	ND[1]	ND
Palmitic acid (C16:0)	13.61	13.82	12.90	14.45
Margaric acid (C17:0)	ND	0.16	ND	ND
Stearic acid (C18:0)	4.98	4.28	3.81	4.53
Arachidic acid (C20:0)	0.40	0.43	ND	0.43
Behenic acid (C22:0)	ND	0.19	ND	ND
Monounsaturated fatty acid (MUFA)				
Palmitoleic acid (C16:1)	0.54	0.32	ND	0.22
Oleic acid (C18:1 n-9)	33.32	34.95	30.18	35.58
Eicosenoic acid (C20:1 n-9)	0.32	0.26	0.42	ND
Polyunsaturated fatty acid (PUFA)				
Linoleic acid (C18:2 n-6)	42.63	42.85	48.53	42.56
Alpha-linolenic acid (C18:3 n-3)	3.78	2.45	4.16	2.24
Eicosapentaenoic acid (C20:5 n-3)	ND	ND	ND	ND
Docosahexaenoic acid (C22:6 n-3)	ND	ND	ND	ND
Total SFA	19.42	19.16	16.71	19.41
Total MUFA	34.18	35.53	30.6	35.8
Total PUFA	46.41	45.3	52.69	44.8
PUFA:SFA ratio[2]	2.39	2.36	3.15	2.31

[1]ND = not detectable.

[2]PUFA:SFA ratio = total PUFA/total SFA.

Adapted from Almeida et al., 2021.
